# Single Coronary Artery from Right Sinus of Valsalva: A Single Center Experience of Seven Cases

**DOI:** 10.1155/2022/1513474

**Published:** 2022-10-20

**Authors:** Kanhai Lalani, M. Sudhakar Rao, Padmakumar Ramachandran, A. J. Ashwal, Abdul U. K. Razak, Tom Devasia, Ganesh Paramasivam, Pankti Parikh

**Affiliations:** ^1^Department of Cardiology, Kasturba Medical College, Manipal, Manipal Academy of Higher Education, Manipal, Karnataka 576104, India; ^2^Department of Endocrinology, St. John's Medical College, St. John's National Academy of Health Sciences, Bangalore, Karnataka 560034, India

## Abstract

**Background:**

Single coronary artery (SCA) is a rare anomaly with a prevalence of 0.024–0.066%. Some anomalies are merely benign anatomical variants, whereas some can result in myocardial ischemia or life-threatening arrhythmia. *Case Presentation*. We described seven cases in which all three major coronaries emerged from the right sinus of Valsalva via a single ostium and supplied the vast majority of the myocardium. A smaller branch arising from the left sinus supplied a modest quantity of myocardium in some of those few cases. These SCA variations do not exactly fit into any existing classification. It is unclear whether we need to modify previous classification systems or newer classification systems.

**Conclusions:**

SCA is divided based on its anomalous course and is usually a benign condition but it may present with cardiovascular complications. Clinicians should be aware of this entity along with the role of CT angiogram in its diagnosis and management.

## 1. Background

Coronary anomalies are usually incidentally diagnosed during coronary angiogram (CAG), CT coronary angiogram (CCTA), or autopsy studies and affect 0.3–5.6% of the general population [1]. These anomalies differ in number, location, coronary origin, and ostium orientation. A single coronary artery (SCA) is defined as “an isolated coronary artery that arises from a single coronary ostium and provides blood supply to the entire myocardium” [2]. They may or may not be associated with structural heart disease. According to the published literature, the prevalence of SCA in the general population is very low, ranging from 0.024 to 0.066% [1, 3]. The majority of SCA are asymptomatic. However, in some cases, severe life-threatening arrhythmias, myocardial infarction, or sudden cardiac death may occur, requiring medical or surgical intervention or coronary revascularisation procedures [3].

### 1.1. Case Presentation

We reviewed 28,148 coronary angiograms performed at the Department of Cardiology, Kasturba Medical College and Hospital in Southern Karnataka, from 2014 to 2020 and reported 7 cases of the single coronary artery from the right sinus, including its variant, implying a prevalence of 0.025 percent in our population. We described seven cases of a single coronary artery that originated from the right sinus of Valsalva via a common ostium and supplied the majority of the myocardium.

#### 1.1.1. Case 1

**Figure 1:**
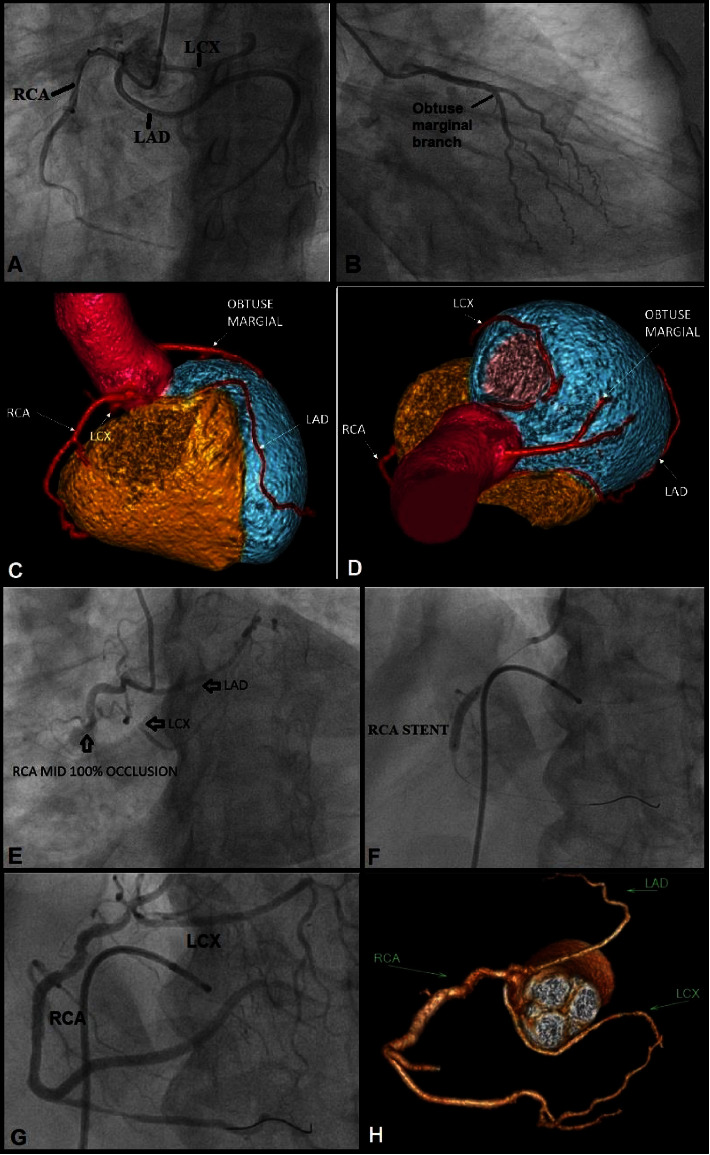
(a): Coronary angiogram showing the origin of all 3 major coronaries from the right coronary sinus via common ostium. (Case 1). (b): CAG showing a branch arising from the left sinus supplying obtuse marginal territory. (Case 1). (c) & (d): 3D volume-rendered reconstructed CT image showing the origin of LAD, LCX, and RCA from the right sinus of Valsalva via common ostium and origin of the small obtuse marginal branch from the left sinus of Valsalva. (Case 1). (e): CAG showing the origin of all 3 major coronaries from the right sinus along with complete occlusion of RCA and 90% occlusion of LCX. (Case 2). (f): Image showing the stent in RCA along with temporary pacemaker lead in the right ventricle. (Case 2). (g): Post angioplasty image showing TIMI-III flow in RCA and LCX. (Case 2). (h): Volume-rendered coronary tree showing the common origin of all 3 major coronaries from the right sinus. (Case 2).

A 59-year-old hypertensive and diabetic female presented with a history of exertional fatigue NYHA (New York Heart Association) class II and nonanginal chest pain for the last 2 years. The electrocardiogram (ECG) and echocardiogram were normal but her treadmill test (TMT) was positive at 8 METS (metabolic equivalents) without any symptoms. The right and left sinus of Valsalva were successfully cannulated on CAG using 5F TIG (Tiger) 110 cms—OPTITORQUE® Diagnostic Catheter (Terumo Corporation, Tokyo, Japan). An angiogram showed a single coronary artery (SCA) originating from a single right coronary ostium from the right sinus of Valsalva. The right coronary artery (RCA), left circumflex artery (LCX), and left anterior descending (LAD) arteries all emerged from the right coronary sinus and shared a common ostium. The right dominant coronary arterial system was observed, with the RCA continuing as the posterior descending artery (PDA), and it was normal. The LAD, LCX, and RCA were all normal (Figure 1(a)). A branch originating from the left sinus which continued laterally over the left ventricle—likely an obtuse marginal branch. The left main coronary artery (LMCA) was not visible (Figure 1(b)). CCTA confirmed the LAD course below the right ventricular outflow tract (subpulmonic) and then through the septum to the anterior interventricular groove, corresponding to Lipton's class R-III-S (Figures 1(c) and 1(d)). Neither SCA nor its branches had a malignant interarterial course. She experienced nonanginal chest pain, for which she was optimally treated with a statin and antihypertensive medications. She was doing well at the one-year follow-up.

#### 1.1.2. Case 2

A 61-year-old diabetic male presented with anginal pain and sweating for 6 hours. The ECG revealed an acute inferior wall myocardial infarction. The systemic examination was unremarkable. Troponin was elevated. CAG revealed an abnormal origin of all three major coronaries, LAD, LCX, and RCA, from the right sinus of Valsalva via a common ostium. The dominant RCA had complete thrombotic occlusion, whereas the LCX ostial and midportion had 90% stenosis. The LAD was normal (Figure 1(e)). He underwent an emergency angioplasty. Following thrombus aspiration, the RCA lesion was stented with a (3 × 23 mm) everolimus-eluting stent (EES) (Figure 1(f)). Balloon angioplasty was performed to the LCX lesion with a (2 × 15 mm) predilatation balloon. TIMI (thrombolysis in myocardial infarction)—3 flow was achieved, hence LCX was not stented (Figure 1(g)). CCTA revealed that all 3 major coronaries originated from the right sinus via a common ostium with an interarterial course of the LAD, corresponding to Lipton's class R-III-B (Figure 1(h)). This course was considered malignant as compression between the aorta and pulmonary artery would lead to sudden cardiac death during vigorous exercise. He was optimally treated with dual antiplatelets, statin, and antianginal drugs. On 1 year follow-up, he was doing well.

#### 1.1.3. Case 3

**Figure 2:**
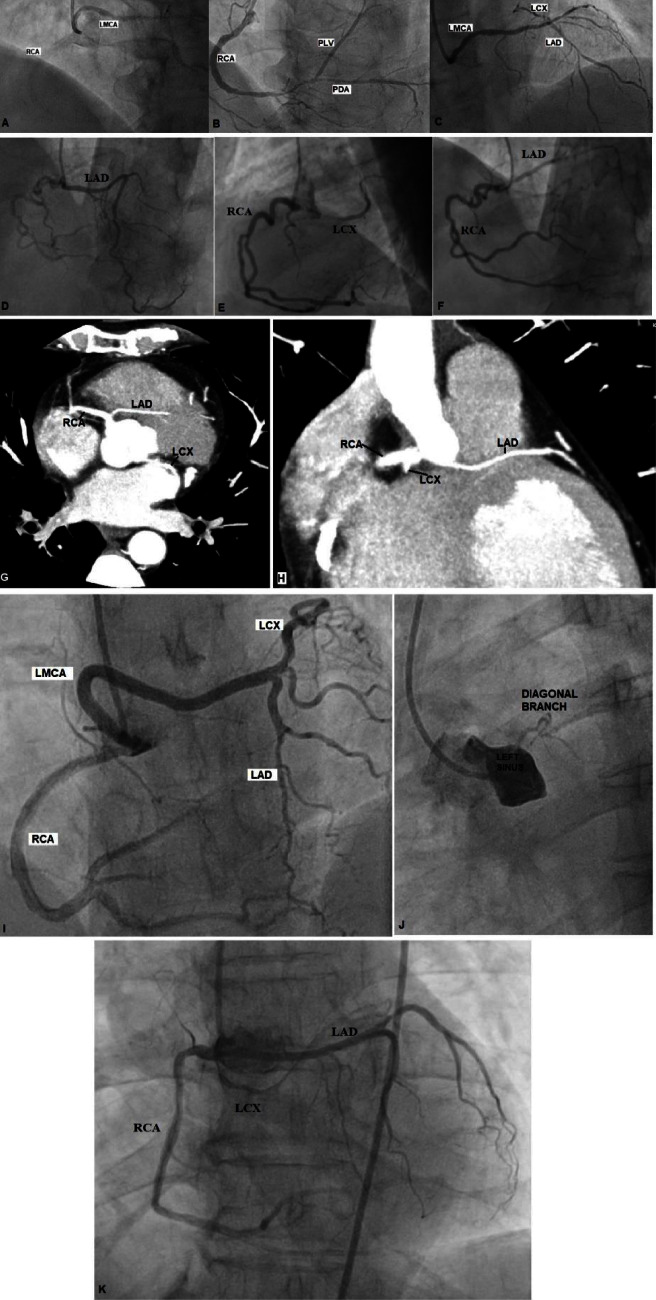
(a)–(c): CAG showing the origin of all 3 major coronaries from the right sinus of Valsalva. Dominant RCA showing 80–90% long tubular stenosis in mid to distal part along with 80% stenosis in proximal PDA. LMCA originates from the right sinus via common ostium with RCA and coursed retroaortic and then divided into LAD and LCX. LAD had long segment calcific 80% stenosis and LCX was distal diseased. (Case 3). (d)–(f): CAG showing single vessel disease with all 3 major coronaries originating from the right sinus via common ostium. LCX ran retroaortic with ostioproximal 95% thrombotic occlusion. LAD had mild plaque. Dominant RCA had 60% stenosis in the mid part. (Case 4). (g) & (h): CT coronary showing all 3 major coronaries LAD, LCX, and RCA arising from the right sinus via the common trunk. RCA showed 90% stenosis in the mid part. (Case 5). (i) & (j): CAG showing anomalous origin of LMCA from the right sinus along with RCA via common ostium. The left main coronary traversed retroaortic towards the left side and divided into the LAD and LCX branches. A small diagonal branch originated from the left sinus. (Case 6). (k): CAG showing all 3 major coronaries LAD, LCX, and RCA originating from the right coronary cusp via the common ostium. (Case 7).

A 71-year-old hypertensive female presented with a one-day history of chest pain. Baseline ECG showed T inversion in inferior and precordial leads. An echocardiogram showed mild to moderate mitral regurgitation, mild concentric hypertrophy, and normal biventricular systolic function. Troponin was elevated. CAG revealed that all three major coronaries originated from the right sinus of Valsalva. Dominant RCA had 80–90% long tubular stenosis in the mid to distal part, and proximal PDA had 80% stenosis (Figures 2(a) and 2(b)). The LMCA originated from the right sinus via a common ostium with the RCA and coursed retroaortically before splitting into the LAD and LCX, corresponding to Lipton's class R-II-P. The LAD had a long segment calcific 80% stenosis, while the LCX was distally diseased (Figures 2(a) and 2(c)). She had percutaneous coronary intervention (PCI) to the RCA, with two EES stents placed in the proximal and distal RCA. She was managed with dual antiplatelets, statin, nitrates, and antihypertensive drugs. On 1 year follow-up, she was doing well.

#### 1.1.4. Case 4

A 60-year-old hypertensive man presented with sudden onset of breathlessness for 1 day. There was a history of orthopnea and paroxysmal nocturnal dyspnea (PND). A physical examination revealed a pulse rate of 140/min and a blood pressure of 190/100 mm Hg. On examination, bilateral crepitation was present. The ECG showed ST-T changes and troponin was elevated (0.331 ng/ml). An echocardiogram showed normal left ventricular function, trivial mitral regurgitation, and mild left ventricular hypertrophy. He was mechanically ventilated. CAG showed single-vessel disease with all 3 major coronaries originating from the right sinus via a common ostium. LAD had mild plaque (Figure 2(d)). LCX ran retroaortic with ostioproximal 95% thrombotic occlusion (Figure 2(e)). The dominant RCA had 60% stenosis in the midpart (Figure 2(f)). He successfully underwent primary PCI to LCX with EES using a 6F Amplatz Right 1 guide. Postprocedure TIMI-3 flow was noted in LCX. CCTA revealed a prepulmonary course of the LAD, corresponding to Lipton's class R-III-A. He was discharged on the third post-op day after being treated with dual antiplatelets, a statin, and diuretics. On 1 year follow-up, he was asymptomatic and doing well.

#### 1.1.5. Case 5

A 62-year-old asymptomatic diabetic male came for gall bladder surgery clearance and his TMT was positive at 7 METS. CAG was performed, which showed all 3 major coronaries LAD, LCX, and RCA arising from the right sinus via the common trunk. The dominant RCA showed 90% stenosis in the midpart. He successfully underwent PCI to the mid-RCA with a sirolimus-eluting stent. A CCTA was performed, which revealed a common trunk originating from the right coronary sinus and from which the LAD, LCX, and RCA originated (Figure 2(g)). The LAD had an interarterial course between the pulmonary trunk and the aortic root with no stenosis, corresponding to Lipton's class R-III-B (Figure 2(h)). The LCX ran posteriorly between the aortic root and the left atrium—a retroaortic course with normal contrast opacification (Figures 2(g) and 2(h)). The dominant RCA showed a patent RCA stent with its normal course. He was discharged on dual antiplatelets, statin, and antidiabetic medications, and he was doing well at the 1-year follow-up.

#### 1.1.6. Case 6

A 46-year-old hypertensive and hyperthyroid female presented with a history of one episode of giddiness but no syncopal attacks. She denied any other cardiac symptoms. Physical and cardiac examinations were both normal. The electrocardiogram showed left ventricular hypertrophy with a strain pattern and the echocardiogram showed apical hypertrophic cardiomyopathy without outflow obstruction with normal ejection fraction. Troponin T level was within normal limits. CAG showed an anomalous origin of the LMCA from the right sinus along with the RCA via a common ostium. The left main coronary artery crossed the aorta retroaortically and divided into the LAD and LCX branches, which supplied the respective territory (Figure 2(i)). The angiographic findings were confirmed by CCTA and classified as Lipton's class R-II-P. The RCA entered the right atrioventricular groove and continued distally as PDA in its normal course. All of the major epicardial coronaries were normal. However, smaller diagonal branch originating from the left sinus was also normal (Figure 2(j)). She was advised of medical management and was doing well after a one-year follow-up.

#### 1.1.7. Case 7

A 68-year-old hypertensive male presented with complaints of weakness in the left upper limb of 1-month duration. Physical and cardiac examinations were both normal. The ECG and echocardiogram were both normal. MRI revealed a subacute infarct in the right precentral gyrus, as well as a subacute infarct in the right centrum semiovale and corona radiata. Carotid doppler showed an echogenic thrombus for a length of 2 cm in the right proximal internal carotid artery (ICA), causing near-complete occlusion. A carotid angiogram revealed 100% thrombotic occlusion of the right proximal ICA. CAG revealed that all 3 major coronaries (LAD, LCX, and RCA) originated from the right coronary cusp via a common ostium (Figure 2(k)), and CCTA revealed Lipton's class R-III-A. The LAD emerged from the right sinus, traversed the left side, and entered the anterior interventricular groove. The LAD was normal, but its diagonal branch showed 70% stenosis. The LCX emerged from the right sinus, travelled retroaortically, and then continued distally in the posterior atrioventricular groove. The dominant RCA arose from the right sinus and continued in its normal anatomical course. He was asymptomatic for his cardiac condition, so he was prescribed single antiplatelet, statin, and antihypertensive medications.

## 2. Discussion

Coronary anomalies are rare and usually diagnosed incidentally during coronary angiography, CT coronary angiography, or autopsy studies. Its prevalence is approximately 0.3–5.6% in the general population [1]. Among these, the single coronary artery is a very rare condition, with an estimated prevalence of 0.024–0.066% based on a published literature [1]. SCA is defined as “an isolated coronary artery that arises from a single coronary ostium and provides blood supply to the entire myocardium” [2]. It may not be associated with other congenital heart diseases [1].

Several classification systems for SCA had been described. The classification for SCA was introduced by Smith, Ogden and Goodyear, Lipton et al., and Shirani and Roberts, to describe the origin and course of SCA. [3–6] None of these classification systems are complete, but they do aid in the classification of various types of SCA. However, Lipton et al. [6] classification system is widely used, and it was later modified by Yamanaka and Hobbs [1] in 1990, based on anatomical distribution, ostial location, and the course of the transverse trunk [1, 6].

We described 7 cases of SCA (∼0.025% prevalence) in which all three major coronaries originated from the right sinus of Valsalva via a common ostium and supplied the majority of the myocardium. This type of anatomy corresponds to the R-II or R-III type of Lipton's classification. Out of 7 cases, two cases had a branch originating from the left sinus supplying the smaller area of the myocardium. Even though there were two coronary ostia in our patient, the entire heart was essentially supplied by the right coronary system. Because these cases did not exactly correspond to any of the aforementioned classification systems, we have considered them as a variant of SCA. Except for the aberrant small branch, this anatomy correlates with Lipton's type R-II or R-III. Even in the literature, it is unclear whether such cases should be classified as single coronary systems or not. A similar type of case was reported by Subban et al. [7] where the RCA and LAD arose from the right sinus and the RCA continued as the LCX distally, along with a small diminutive diagonal branch from the left sinus. They had described such a case as a single coronary system as the majority of the heart was supplied by the right coronary system [7]. We need further data to update these classification systems to include such cases.

A coronary angiogram can clearly identify anomalies in 53% of cases only. CT angiography can precisely delineate anatomic abnormality, acute angle take-off, the number of Ostia, the course of the coronaries, and their relationship to the great arteries [8, 9]. A 64-slice CT angiogram has a sensitivity of 89% and a specificity of 96% for detecting coronary stenosis [10].

The majority of individuals with SCA are asymptomatic or have nonspecific symptoms; nevertheless, certain variants of SCA can cause angina, myocardial ischemia, syncope, and severe life-threatening arrhythmias. Sudden cardiac death can be a presenting feature if SCA courses between the pulmonary artery and the aorta [3]. Our 2 cases had such type of interarterial course of LAD. According to Shirani and Roberts, 15 percent of SCA cases develop angina and ischemia as a result of aberrant coronary anatomy rather than coronary artery diseases [3].

Anomalous LAD originating from the right sinus can take one of four paths: septal, anterior free wall, retroaortic, or interarterial. The septal course is mostly benign and no cases of sudden cardiac death have been reported [11].

Although anomalous coronaries are rare, they continue to be at risk for CAD and should be treated in the same way as native coronaries [12]. Hutchins et al. showed that the abnormal tortuous course of the proximal part of anomalous coronaries is susceptible to lipid accumulation, predisposing them to increased atherosclerosis [13]. Anomalous LCX from the right sinus has a higher risk of stenosis compared to normal origin [14].

Considering the wide range of anatomical presentations of SCA, a comprehensive strategy involving cardiologists and cardiac surgeons should be considered while selecting the most appropriate treatment. A severe symptomatic patient with CAD requires angioplasty or coronary artery bypass surgery, whereas minimally symptomatic patients with no significant CAD require medical management [15]. Patients who present with syncope of unknown origin, malignant arrhythmia, or resuscitated sudden cardiac arrest due to an abnormal interarterial course are usually advised to undergo surgical management via aortocoronary bypass, coronary reimplantation, or unroofing of an intramural coronary segment [16]. Surgery is recommended if there is evidence of myocardial ischemia, even in asymptomatic patients with an interarterial course; it is also recommended for patients under 30–35 years of age, even if the stress test is negative. The risk of sudden cardiac death in elderly people declines with age and is exceedingly low, possibly due to the hardening of the aortic wall in the elderly population [16]. In our series, 4 out of 7 patients who underwent coronary artery stenting had no symptoms on follow-up. The other 3 patients had no coronary artery disease and were followed up periodically as they were asymptomatic.

## 3. Conclusions

SCA is one of the rarest coronary anomalies. It is classified based on its anomalous course. SCA is usually a benign condition but it may present with myocardial ischemia, life-threatening arrhythmia, or sudden cardiac death. Clinicians and surgeons should be aware of this entity along with the role of CT angiogram in its diagnosis and management.

## Data Availability

Not applicable.

## Abbreviations

SCA: Single coronary artery
CTCA: Coronary computed tomography angiography
CAG: Coronary angiogram
NYHA: New York Heart Association
METS: Metabolic equivalents
TIMI: Thrombolysis in myocardial infarction
LAD: Left anterior descending artery
RCA: Right coronary artery
LCx: Left circumflex artery
LMCA: Left main coronary artery
EES: Everolimus-eluting stent
TMT: Treadmill test
ICA: Internal carotid artery
MRI: Magnetic resonance imaging.
